# Supplementary material for: Molecular Dynamics in the Ventral Tegmental Area during Chronic Pain-Induced Negative Affect
Source: bioRxiv. 2025 Jul 4:2025.07.01.662604. Preprint. [Version 1] doi: 10.1101/2025.07.01.662604 (PMC12236664; doi:10.1101/2025.07.01.662604)
Supplement: Supplement 1 — Supplementary Figure 1. pSNL surgery causes long-lasting allodynia. Violin plot depicts individual animal paw withdrawal threshold values in male and female mice from sham and pSNL-exposed groups (interaction F(2,151)=39.46, ****p<0.0001). Both groups exhibited hypersensitivity at WK1 compared to baseline (****p<0.0001) to different extents (**p=0.0030). While pSNL mice continued to show hypersensitivity at WK4 (**p<0001), sham-exposed mice returned to withdrawal thresholds similar to baseline ****p<0.0001). Sample sizes are indicated in parentheses (Sham male, Sham female; pSNL male, pSNL female). Solid lines in violin plots depict median and dashed lines depict quartiles. Supplementary Figure 2. Early Imbalance in VTA Neuromodulation at WK1 pSNL. Bar graph depicts the relative abundance of various proteins normalized to sham-operated mice (multiple unpaired t-tests). Included are enzymes involved in endocannabinoid (eCB) metabolism (fatty acid amide hydrolase (FAAH) and alpha/beta-hydrolase domain containing 6 (ABHD6)) and eCB synthesis (monoacylglycerol lipase (MGLL) and diacylglycerol lipase alpha (DAGLA)). Also analyzed are proteins important for anandamide production (N-acyl-phosphatidylethanolamine phospholipase D (NAPEPLD), and experience dependent synaptic plasticity (KS6KA5 (MSK1), and KAP3 (Prkar2b)). All data are expressed as mean ± SEM. Supplementary Figure 3. Cytosolic Expression Patterns of TH+ and Kv7.2–5 Among VTA cells. High magnification confocal photomicrographs of the contralateral VTA region from male mice, divided into sham-operated (left two panels) and pSNL (right panel) groups, at four weeks post-surgery. Images depict co-localization of TH (red), Kv7.2–5 (green, presented in descending order), and nuclear DAPI staining (blue). Magnified images focusing on individual neurons (far right) show only Kv7 and DAPI staining. Images underwent rolling-ball background subtraction, linear contrast adjustment, and resizing for display. Scale bars repr [file media-1.pdf]

Supplemental Figures

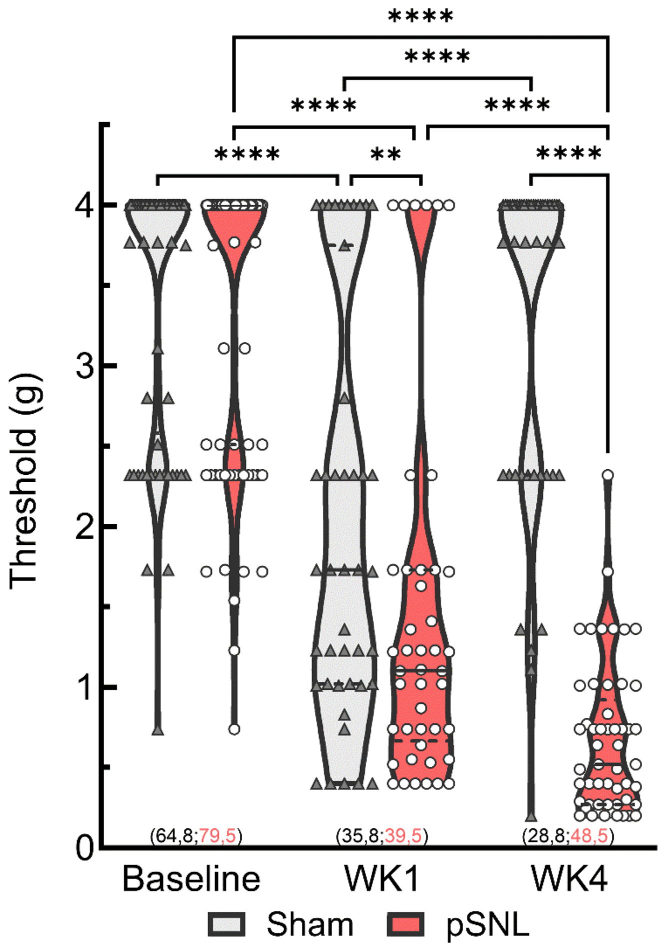

Supplementary Figure 1. pSNL surgery causes long-lasting allodynia. Violin plot depicts individual animal paw withdrawal threshold values in male and female mice from sham and pSNL-exposed groups (interaction $F_{(2,151)}$=39.46, ****p<0.0001). Both groups exhibited hypersensitivity at WK1 compared to baseline (****p<0.0001) to different extents (**p=0.0030). While pSNL mice continued to show hypersensitivity at WK4 (**p<0001), sham-exposed mice returned to withdrawal thresholds similar to baseline ****p<0.0001). Sample sizes are indicated in parentheses (Sham male, Sham female; pSNL male, pSNL female). Solid lines in violin plots depict median and dashed lines depict quartiles.

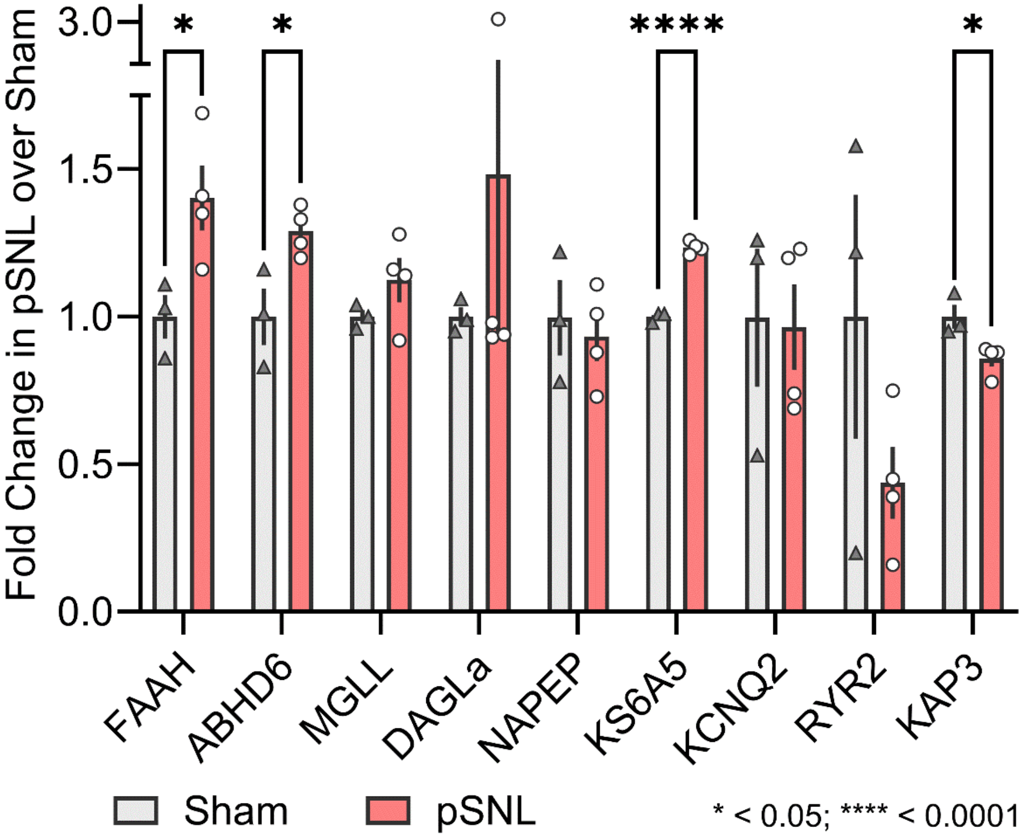

Supplementary Figure 2. Early Imbalance in VTA Neuromodulation at WK1 pSNL. Bar graph depicts the relative abundance of various proteins normalized to sham-operated mice (multiple unpaired t-tests). Included are enzymes involved in endocannabinoid (eCB) metabolism (fatty acid amide hydrolase (FAAH) and alpha/beta-hydrolase domain containing 6 (ABHD6)) and eCB synthesis (monoacylglycerol lipase (MGLL) and diacylglycerol lipase alpha (DAGLA)). Also analyzed are proteins important for anandamide production (N-acyl-phosphatidylethanolamine phospholipase D (NAPEPLD), and experience dependent synaptic plasticity (KS6KA5 (MSK1), and KAP3 (Prkar2b)). All data are expressed as mean ± SEM.

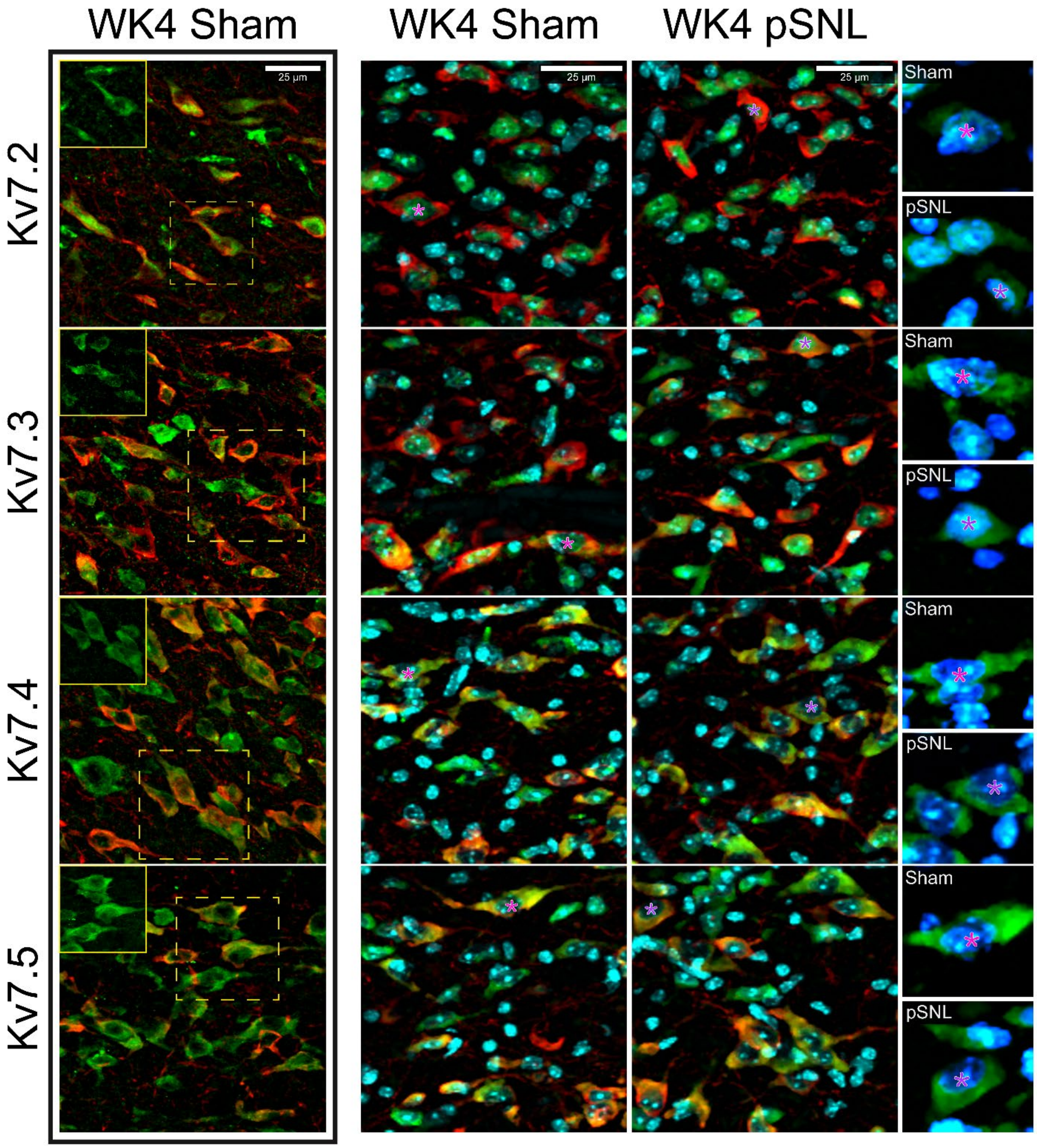

Supplementary Figure 3. Cytosolic Expression Patterns of TH+ and Kv7.2-5 Among VTA cells. High magnification confocal photomicrographs of the contralateral VTA region from male mice, divided into sham-operated (*left two panels*) and pSNL (*right panel*) groups, at four weeks post-surgery. Images depict co-localization of TH (*red*), Kv7.2-5 (*green*, presented in descending order), and nuclear DAPI staining (*blue*). Magnified images focusing on individual neurons (*far right*) show only Kv7 and DAPI staining. Images underwent rolling-ball background subtraction, linear contrast adjustment, and resizing for display. Scale bars represent 25 µm and asterisks represent the nucleus.
